# Supplementary material for: Spatial inter-centromeric interactions facilitated the emergence of evolutionary new centromeres
Source: eLife. 2020 May 29;9:e58556. doi: 10.7554/eLife.58556 (PMC7292649; doi:10.7554/eLife.58556)
Supplement: Supplementary file 1. [file elife-58556-supp1.pptx]

## Slide 1
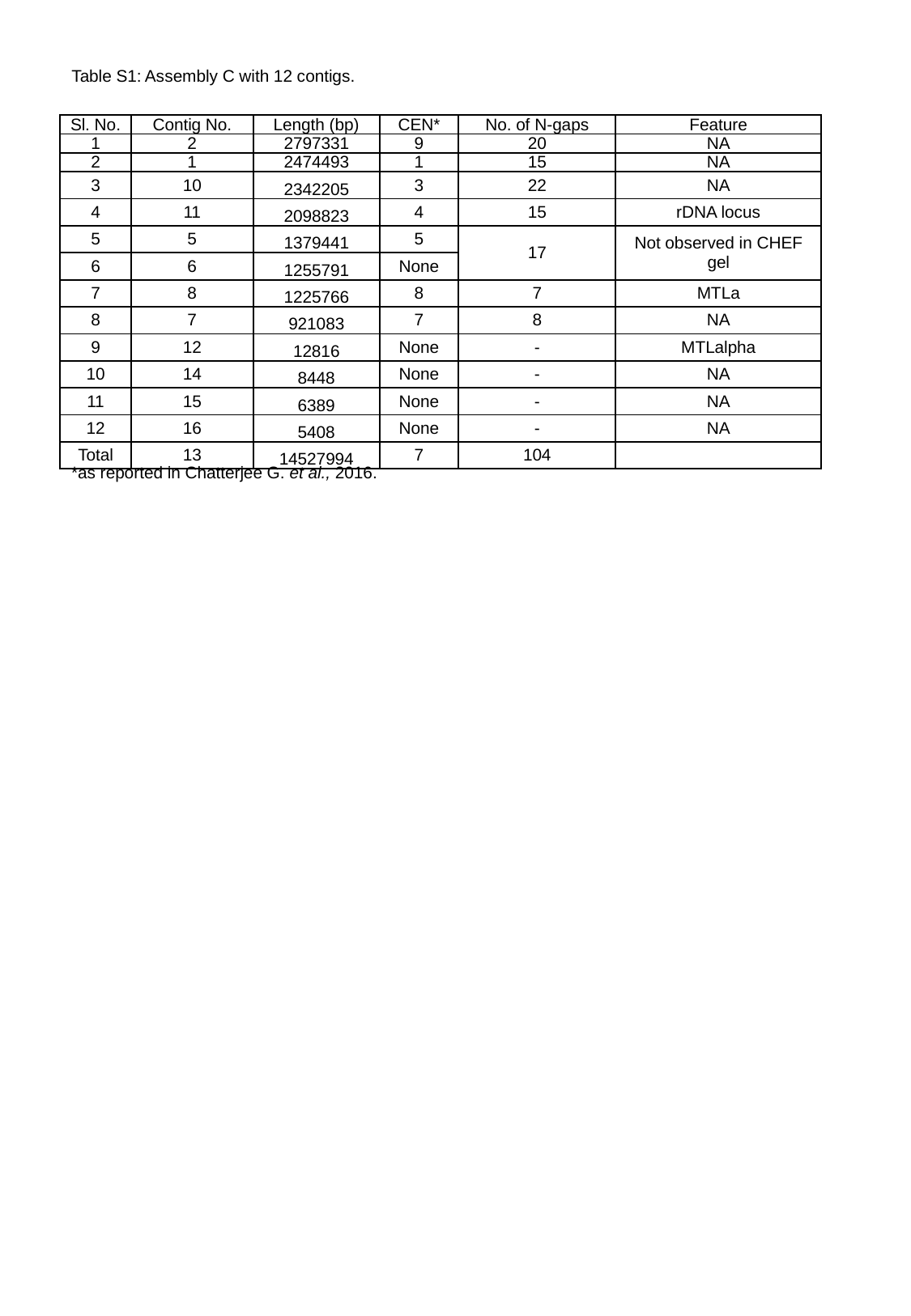

Table S1: Assembly C with 12 contigs.
| Sl. No. | Contig No. | Length (bp) | CEN\* | No. of N-gaps | Feature |
| --- | --- | --- | --- | --- | --- |
| 1 | 2 | 2797331 | 9 | 20 | NA |
| 2 | 1 | 2474493 | 1 | 15 | NA |
| 3 | 10 | 2342205 | 3 | 22 | NA |
| 4 | 11 | 2098823 | 4 | 15 | rDNA locus |
| 5 | 5 | 1379441 | 5 | 17 | Not observed in CHEF gel |
| 6 | 6 | 1255791 | None | | |
| 7 | 8 | 1225766 | 8 | 7 | MTLa |
| 8 | 7 | 921083 | 7 | 8 | NA |
| 9 | 12 | 12816 | None | - | MTLalpha |
| 10 | 14 | 8448 | None | - | NA |
| 11 | 15 | 6389 | None | - | NA |
| 12 | 16 | 5408 | None | - | NA |
| Total | 13 | 14527994 | 7 | 104 | |
*as reported in Chatterjee G. et al., 2016.
