# Supplementary material for: Spatial inter-centromeric interactions facilitated the emergence of evolutionary new centromeres
Source: eLife. 2020 May 29;9:e58556. doi: 10.7554/eLife.58556 (PMC7292649; doi:10.7554/eLife.58556)
Supplement: Supplementary file 2. [file elife-58556-supp2.pptx]

## Slide 1
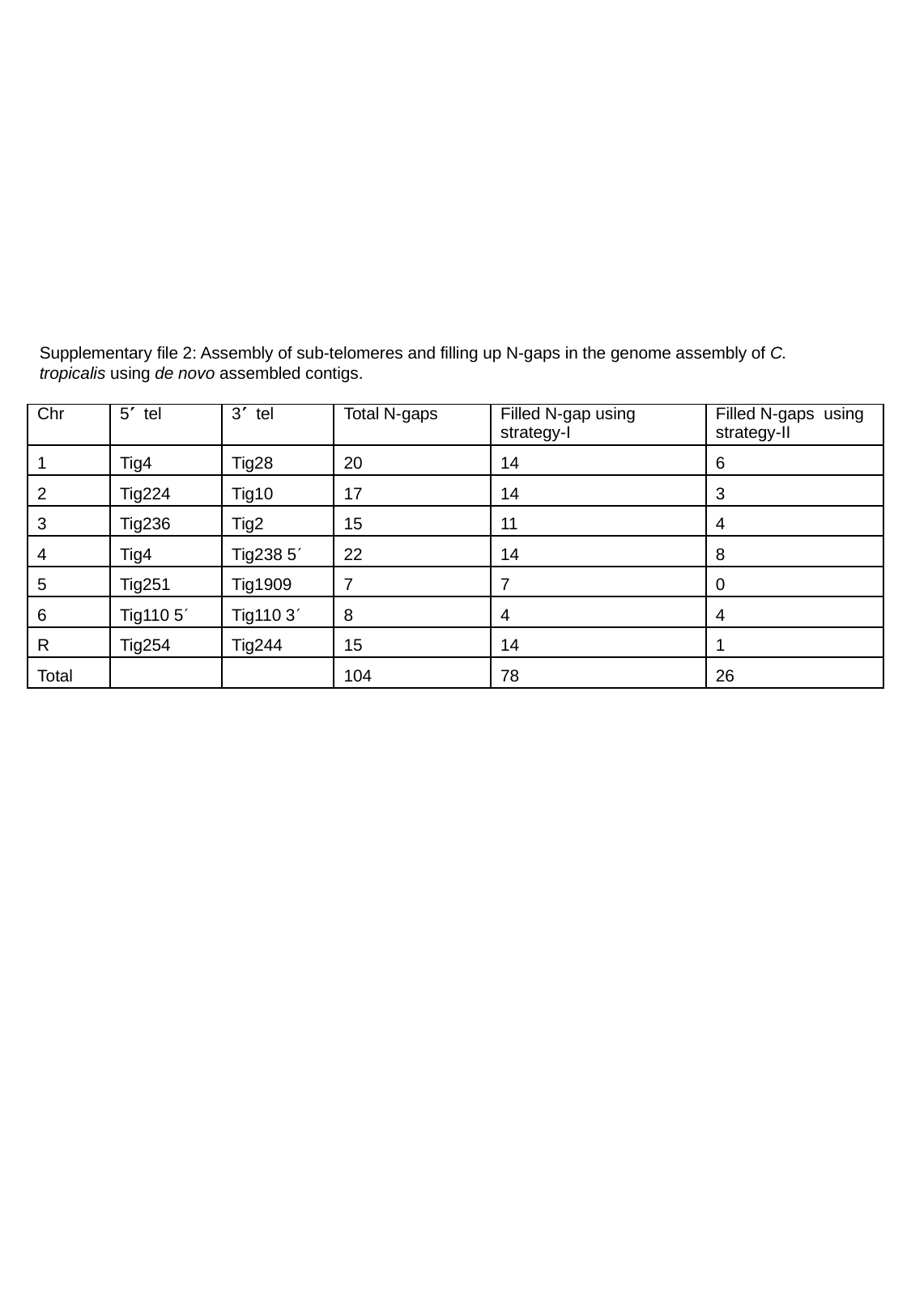

Supplementary file 2: Assembly of sub-telomeres and filling up N-gaps in the genome assembly of C. tropicalis using de novo assembled contigs.
| Chr | 5′ tel | 3′ tel | Total N-gaps | Filled N-gap using strategy-I | Filled N-gaps using strategy-II |
| --- | --- | --- | --- | --- | --- |
| 1 | Tig4 | Tig28 | 20 | 14 | 6 |
| 2 | Tig224 | Tig10 | 17 | 14 | 3 |
| 3 | Tig236 | Tig2 | 15 | 11 | 4 |
| 4 | Tig4 | Tig238 5′ | 22 | 14 | 8 |
| 5 | Tig251 | Tig1909 | 7 | 7 | 0 |
| 6 | Tig110 5′ | Tig110 3′ | 8 | 4 | 4 |
| R | Tig254 | Tig244 | 15 | 14 | 1 |
| Total | | | 104 | 78 | 26 |
