# Supplementary material for: Spatial inter-centromeric interactions facilitated the emergence of evolutionary new centromeres
Source: eLife. 2020 May 29;9:e58556. doi: 10.7554/eLife.58556 (PMC7292649; doi:10.7554/eLife.58556)
Supplement: Supplementary file 3. [file elife-58556-supp3.pptx]

## Slide 1
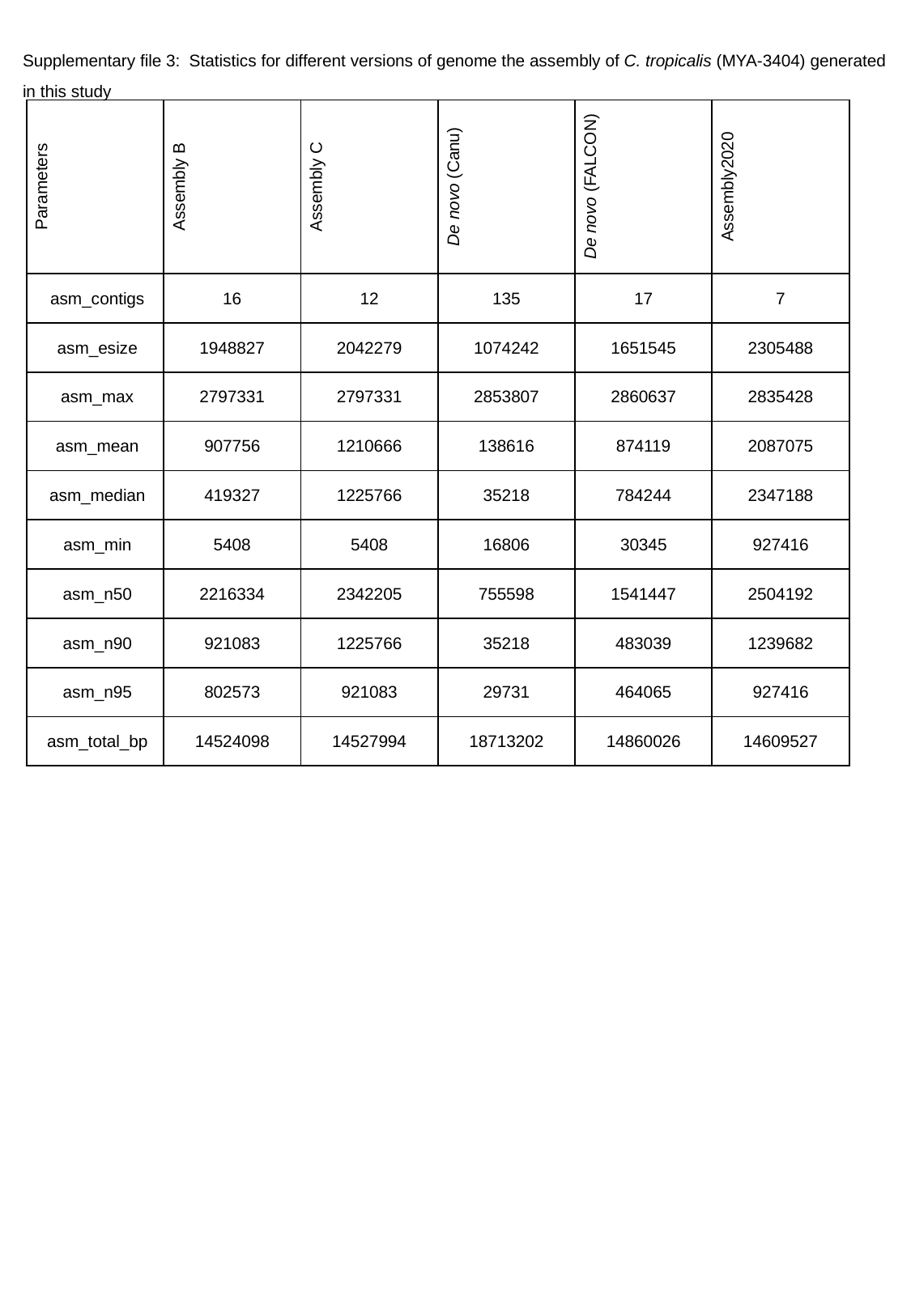

Supplementary file 3: Statistics for different versions of genome the assembly of C. tropicalis (MYA-3404) generated in this study
| Parameters | Assembly B | Assembly C | De novo (Canu) | De novo (FALCON) | Assembly2020 |
| --- | --- | --- | --- | --- | --- |
| asm\_contigs | 16 | 12 | 135 | 17 | 7 |
| asm\_esize | 1948827 | 2042279 | 1074242 | 1651545 | 2305488 |
| asm\_max | 2797331 | 2797331 | 2853807 | 2860637 | 2835428 |
| asm\_mean | 907756 | 1210666 | 138616 | 874119 | 2087075 |
| asm\_median | 419327 | 1225766 | 35218 | 784244 | 2347188 |
| asm\_min | 5408 | 5408 | 16806 | 30345 | 927416 |
| asm\_n50 | 2216334 | 2342205 | 755598 | 1541447 | 2504192 |
| asm\_n90 | 921083 | 1225766 | 35218 | 483039 | 1239682 |
| asm\_n95 | 802573 | 921083 | 29731 | 464065 | 927416 |
| asm\_total\_bp | 14524098 | 14527994 | 18713202 | 14860026 | 14609527 |
