# Supplementary material for: Spatial inter-centromeric interactions facilitated the emergence of evolutionary new centromeres
Source: eLife. 2020 May 29;9:e58556. doi: 10.7554/eLife.58556 (PMC7292649; doi:10.7554/eLife.58556)
Supplement: Supplementary file 4. [file elife-58556-supp4.pptx]

## Slide 1
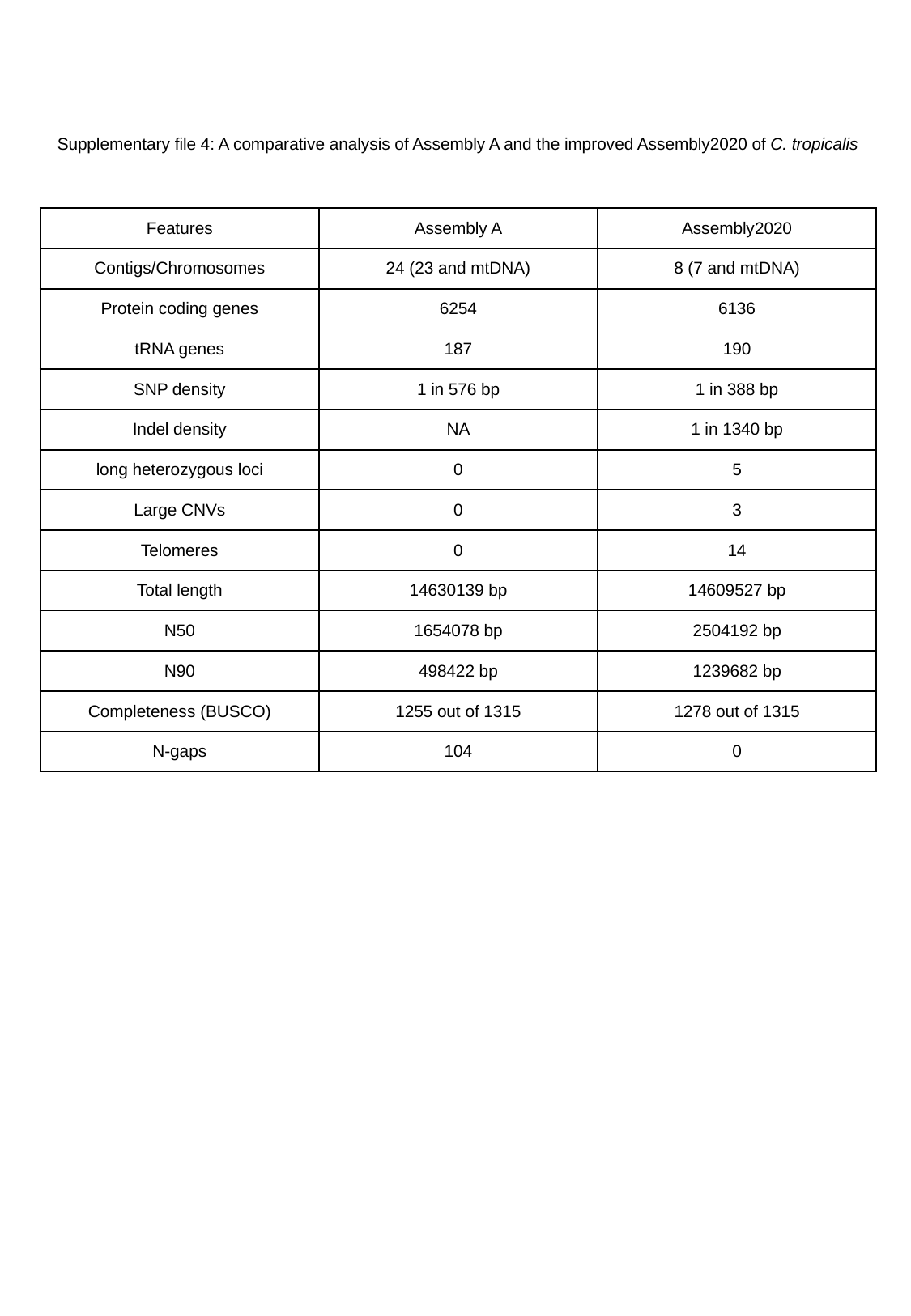

Supplementary file 4: A comparative analysis of Assembly A and the improved Assembly2020 of C. tropicalis
| Features | Assembly A | Assembly2020 |
| --- | --- | --- |
| Contigs/Chromosomes | 24 (23 and mtDNA) | 8 (7 and mtDNA) |
| Protein coding genes | 6254 | 6136 |
| tRNA genes | 187 | 190 |
| SNP density | 1 in 576 bp | 1 in 388 bp |
| Indel density | NA | 1 in 1340 bp |
| long heterozygous loci | 0 | 5 |
| Large CNVs | 0 | 3 |
| Telomeres | 0 | 14 |
| Total length | 14630139 bp | 14609527 bp |
| N50 | 1654078 bp | 2504192 bp |
| N90 | 498422 bp | 1239682 bp |
| Completeness (BUSCO) | 1255 out of 1315 | 1278 out of 1315 |
| N-gaps | 104 | 0 |
