# Supplementary material for: Spatial inter-centromeric interactions facilitated the emergence of evolutionary new centromeres
Source: eLife. 2020 May 29;9:e58556. doi: 10.7554/eLife.58556 (PMC7292649; doi:10.7554/eLife.58556)
Supplement: Supplementary file 5. [file elife-58556-supp5.pptx]

## Slide 1
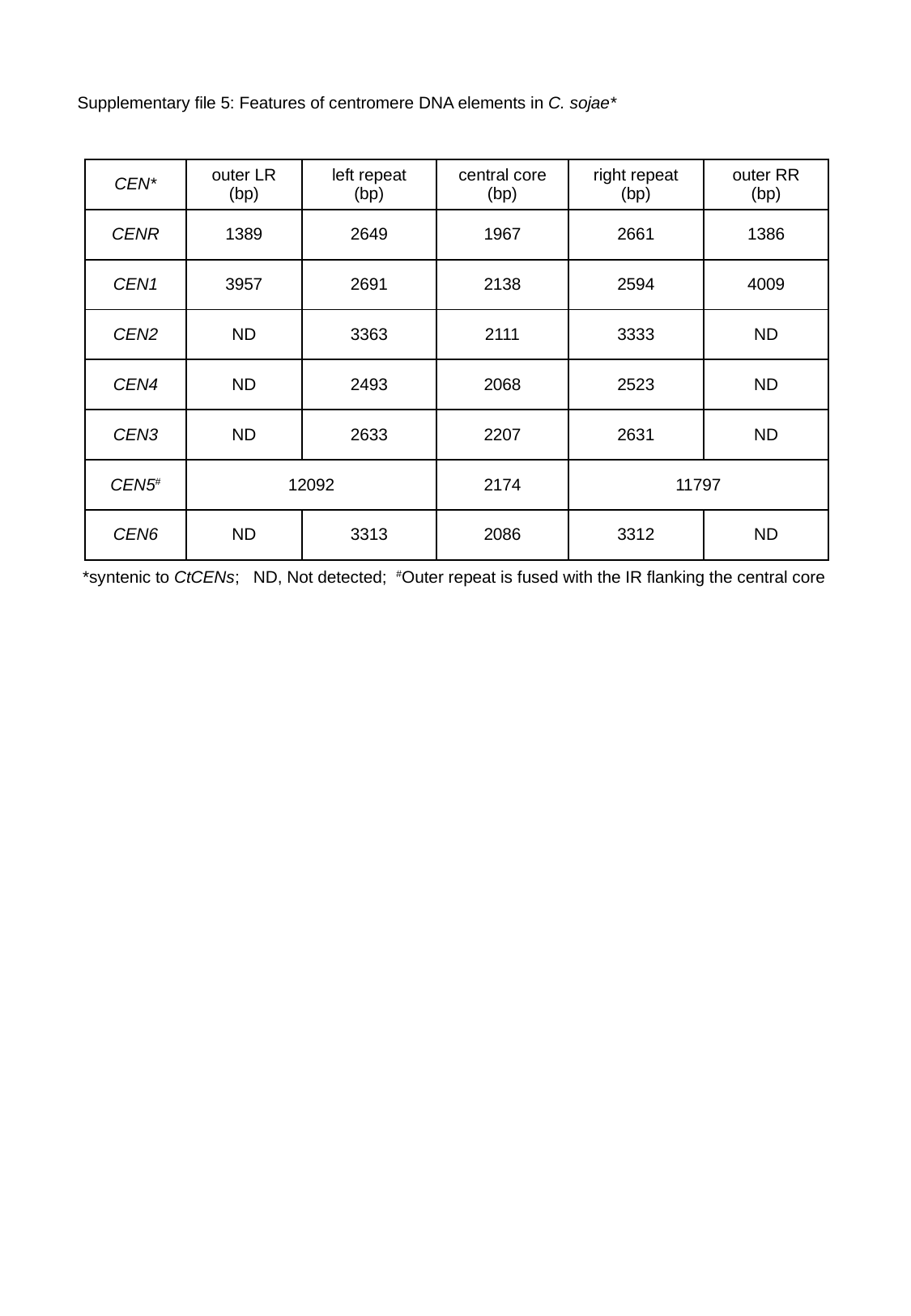

Supplementary file 5: Features of centromere DNA elements in C. sojae*
| CEN\* | outer LR (bp) | left repeat (bp) | central core (bp) | right repeat (bp) | outer RR (bp) |
| --- | --- | --- | --- | --- | --- |
| CENR | 1389 | 2649 | 1967 | 2661 | 1386 |
| CEN1 | 3957 | 2691 | 2138 | 2594 | 4009 |
| CEN2 | ND | 3363 | 2111 | 3333 | ND |
| CEN4 | ND | 2493 | 2068 | 2523 | ND |
| CEN3 | ND | 2633 | 2207 | 2631 | ND |
| CEN5# | 12092 | | 2174 | 11797 | |
| CEN6 | ND | 3313 | 2086 | 3312 | ND |
*syntenic to CtCENs; ND, Not detected; #Outer repeat is fused with the IR flanking the central core
