# Supplementary material for: Spatial inter-centromeric interactions facilitated the emergence of evolutionary new centromeres
Source: eLife. 2020 May 29;9:e58556. doi: 10.7554/eLife.58556 (PMC7292649; doi:10.7554/eLife.58556)
Supplement: Supplementary file 6. [file elife-58556-supp6.pptx]

## Slide 1
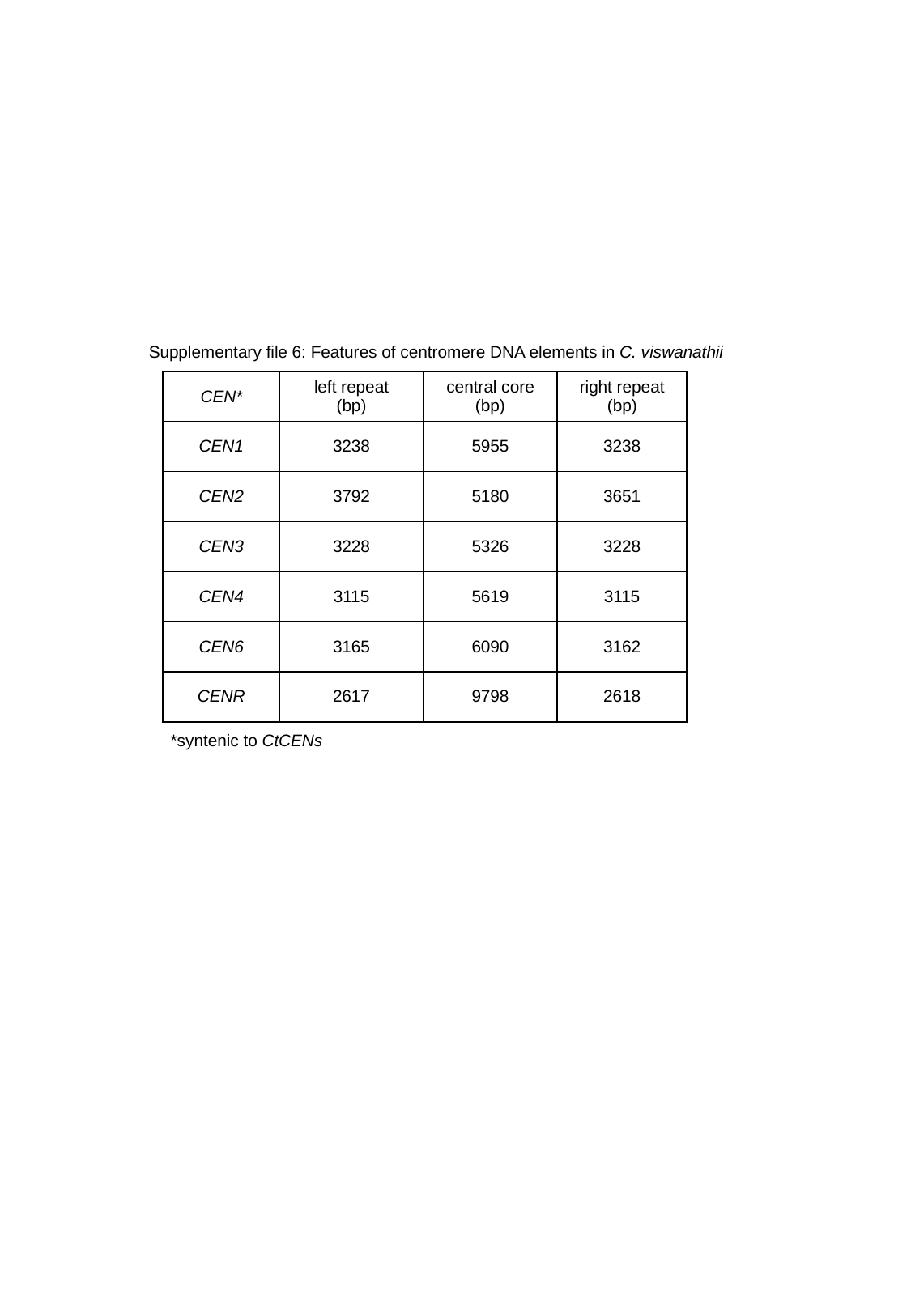

Supplementary file 6: Features of centromere DNA elements in C. viswanathii
| CEN\* | left repeat (bp) | central core (bp) | right repeat (bp) |
| --- | --- | --- | --- |
| CEN1 | 3238 | 5955 | 3238 |
| CEN2 | 3792 | 5180 | 3651 |
| CEN3 | 3228 | 5326 | 3228 |
| CEN4 | 3115 | 5619 | 3115 |
| CEN6 | 3165 | 6090 | 3162 |
| CENR | 2617 | 9798 | 2618 |
*syntenic to CtCENs
