# Supplementary material for: Spatial inter-centromeric interactions facilitated the emergence of evolutionary new centromeres
Source: eLife. 2020 May 29;9:e58556. doi: 10.7554/eLife.58556 (PMC7292649; doi:10.7554/eLife.58556)
Supplement: Supplementary file 7. [file elife-58556-supp7.pptx]

## Slide 1
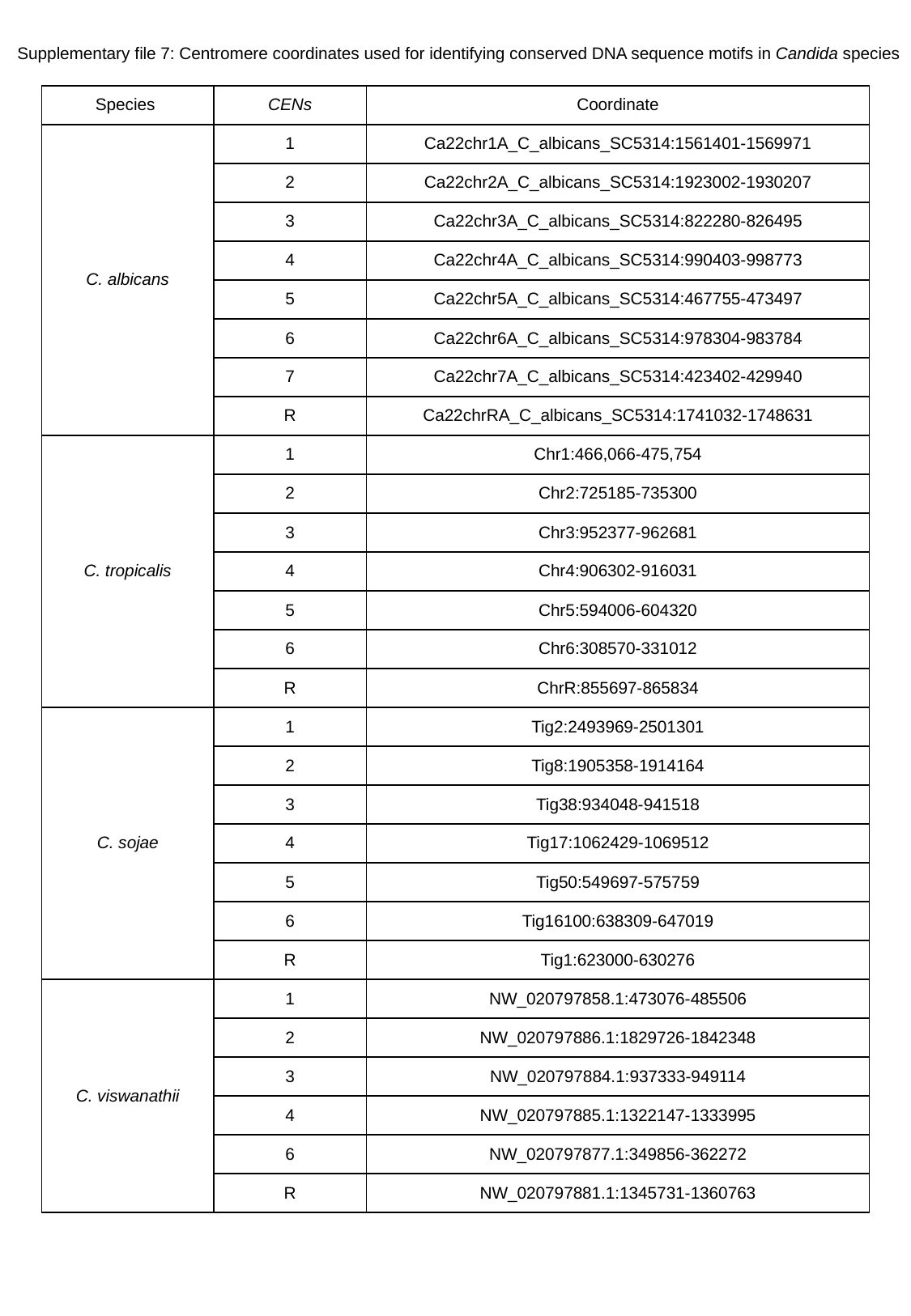

Supplementary file 7: Centromere coordinates used for identifying conserved DNA sequence motifs in Candida species
| Species | CENs | Coordinate |
| --- | --- | --- |
| C. albicans | 1 | Ca22chr1A\_C\_albicans\_SC5314:1561401-1569971 |
| | 2 | Ca22chr2A\_C\_albicans\_SC5314:1923002-1930207 |
| | 3 | Ca22chr3A\_C\_albicans\_SC5314:822280-826495 |
| | 4 | Ca22chr4A\_C\_albicans\_SC5314:990403-998773 |
| | 5 | Ca22chr5A\_C\_albicans\_SC5314:467755-473497 |
| | 6 | Ca22chr6A\_C\_albicans\_SC5314:978304-983784 |
| | 7 | Ca22chr7A\_C\_albicans\_SC5314:423402-429940 |
| | R | Ca22chrRA\_C\_albicans\_SC5314:1741032-1748631 |
| C. tropicalis | 1 | Chr1:466,066-475,754 |
| | 2 | Chr2:725185-735300 |
| | 3 | Chr3:952377-962681 |
| | 4 | Chr4:906302-916031 |
| | 5 | Chr5:594006-604320 |
| | 6 | Chr6:308570-331012 |
| | R | ChrR:855697-865834 |
| C. sojae | 1 | Tig2:2493969-2501301 |
| | 2 | Tig8:1905358-1914164 |
| | 3 | Tig38:934048-941518 |
| | 4 | Tig17:1062429-1069512 |
| | 5 | Tig50:549697-575759 |
| | 6 | Tig16100:638309-647019 |
| | R | Tig1:623000-630276 |
| C. viswanathii | 1 | NW\_020797858.1:473076-485506 |
| | 2 | NW\_020797886.1:1829726-1842348 |
| | 3 | NW\_020797884.1:937333-949114 |
| | 4 | NW\_020797885.1:1322147-1333995 |
| | 6 | NW\_020797877.1:349856-362272 |
| | R | NW\_020797881.1:1345731-1360763 |

## Slide 2
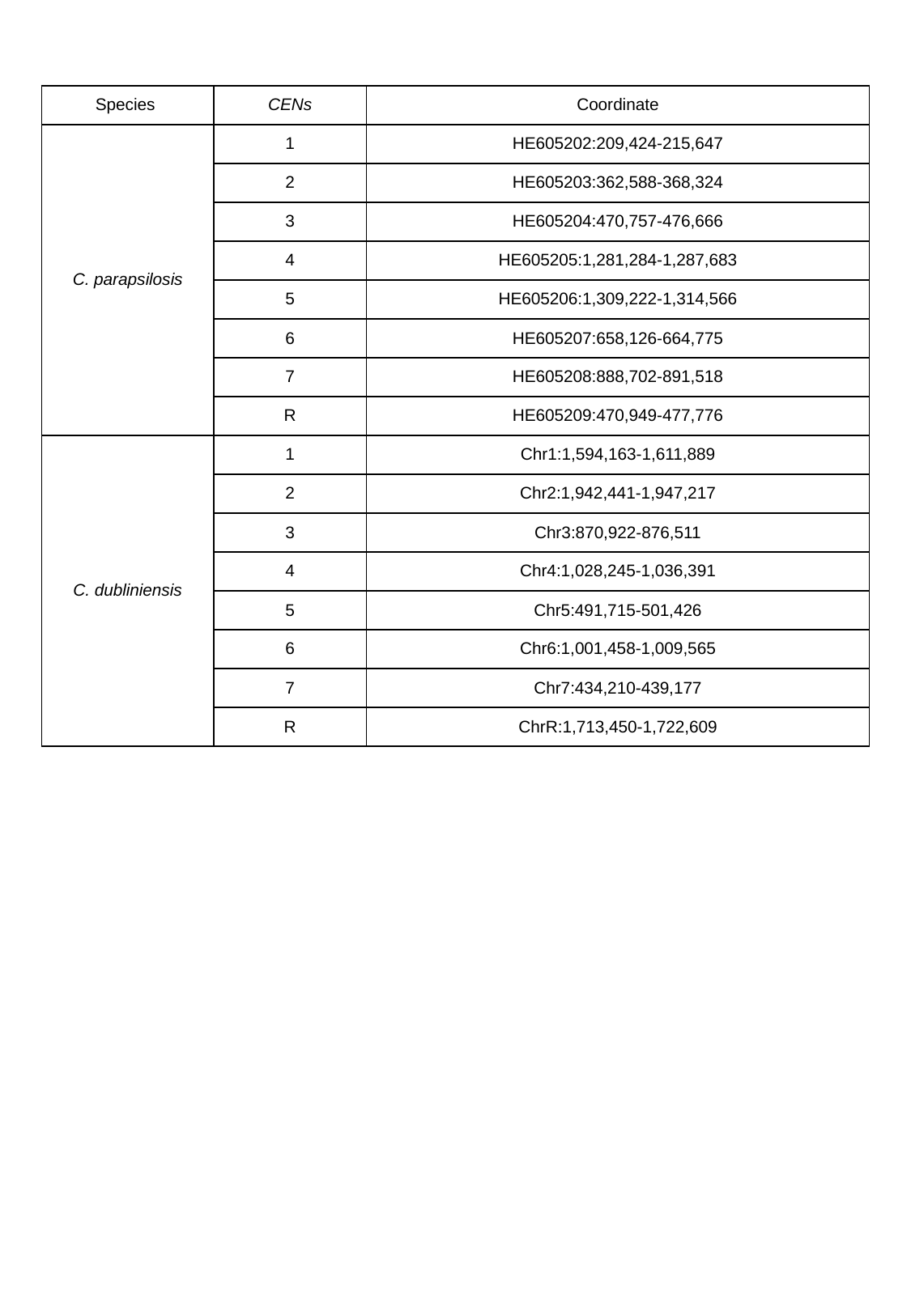

| Species | CENs | Coordinate |
| --- | --- | --- |
| C. parapsilosis | 1 | HE605202:209,424-215,647 |
| | 2 | HE605203:362,588-368,324 |
| | 3 | HE605204:470,757-476,666 |
| | 4 | HE605205:1,281,284-1,287,683 |
| | 5 | HE605206:1,309,222-1,314,566 |
| | 6 | HE605207:658,126-664,775 |
| | 7 | HE605208:888,702-891,518 |
| | R | HE605209:470,949-477,776 |
| C. dubliniensis | 1 | Chr1:1,594,163-1,611,889 |
| | 2 | Chr2:1,942,441-1,947,217 |
| | 3 | Chr3:870,922-876,511 |
| | 4 | Chr4:1,028,245-1,036,391 |
| | 5 | Chr5:491,715-501,426 |
| | 6 | Chr6:1,001,458-1,009,565 |
| | 7 | Chr7:434,210-439,177 |
| | R | ChrR:1,713,450-1,722,609 |
