# Supplementary material for: Spatial inter-centromeric interactions facilitated the emergence of evolutionary new centromeres
Source: eLife. 2020 May 29;9:e58556. doi: 10.7554/eLife.58556 (PMC7292649; doi:10.7554/eLife.58556)
Supplement: Supplementary file 8. [file elife-58556-supp8.pptx]

## Slide 1
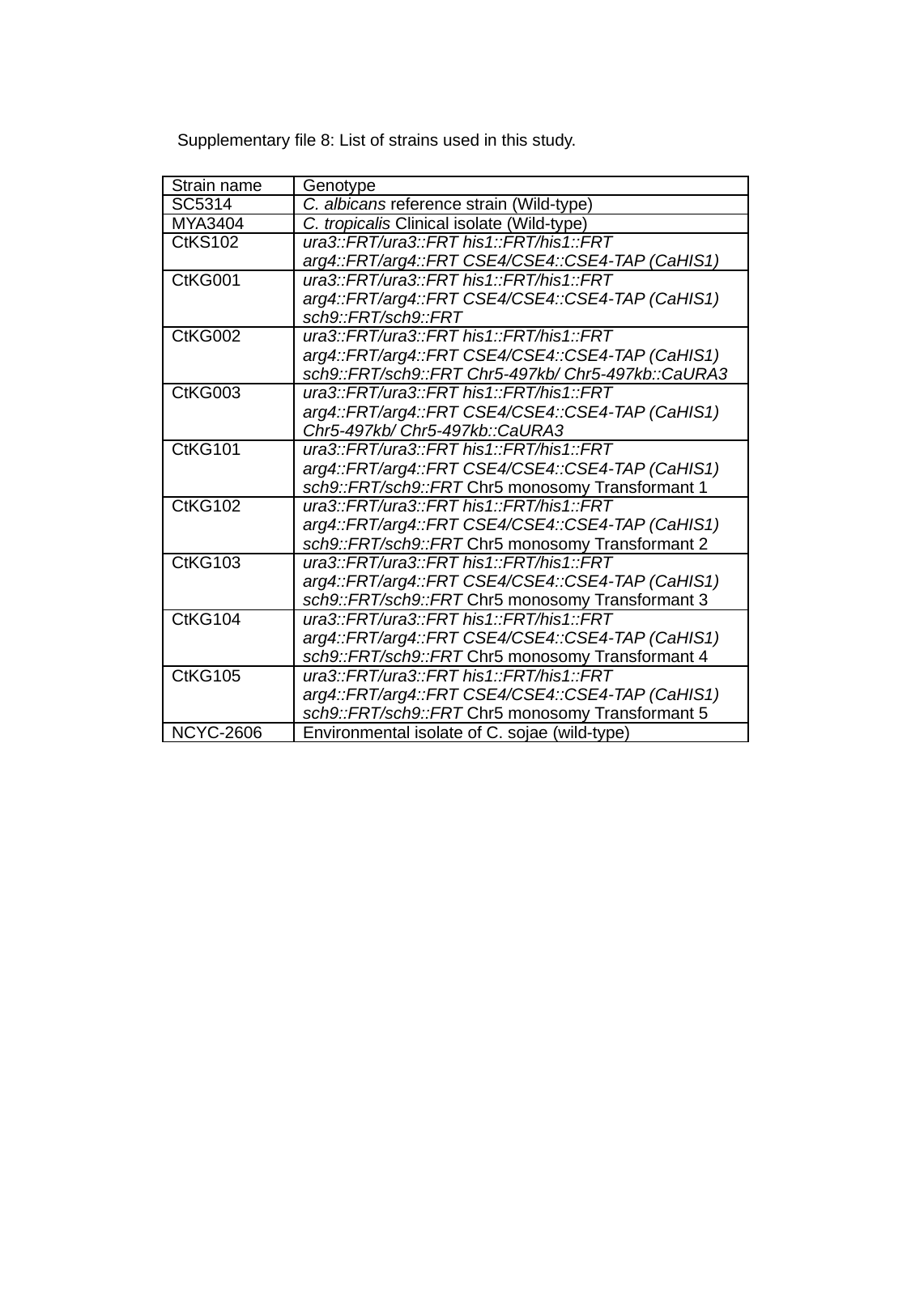

Supplementary file 8: List of strains used in this study.
| Strain name | Genotype |
| --- | --- |
| SC5314 | C. albicans reference strain (Wild-type) |
| MYA3404 | C. tropicalis Clinical isolate (Wild-type) |
| CtKS102 | ura3::FRT/ura3::FRT his1::FRT/his1::FRT arg4::FRT/arg4::FRT CSE4/CSE4::CSE4-TAP (CaHIS1) |
| CtKG001 | ura3::FRT/ura3::FRT his1::FRT/his1::FRT arg4::FRT/arg4::FRT CSE4/CSE4::CSE4-TAP (CaHIS1) sch9::FRT/sch9::FRT |
| CtKG002 | ura3::FRT/ura3::FRT his1::FRT/his1::FRT arg4::FRT/arg4::FRT CSE4/CSE4::CSE4-TAP (CaHIS1) sch9::FRT/sch9::FRT Chr5-497kb/ Chr5-497kb::CaURA3 |
| CtKG003 | ura3::FRT/ura3::FRT his1::FRT/his1::FRT arg4::FRT/arg4::FRT CSE4/CSE4::CSE4-TAP (CaHIS1) Chr5-497kb/ Chr5-497kb::CaURA3 |
| CtKG101 | ura3::FRT/ura3::FRT his1::FRT/his1::FRT arg4::FRT/arg4::FRT CSE4/CSE4::CSE4-TAP (CaHIS1) sch9::FRT/sch9::FRT Chr5 monosomy Transformant 1 |
| CtKG102 | ura3::FRT/ura3::FRT his1::FRT/his1::FRT arg4::FRT/arg4::FRT CSE4/CSE4::CSE4-TAP (CaHIS1) sch9::FRT/sch9::FRT Chr5 monosomy Transformant 2 |
| CtKG103 | ura3::FRT/ura3::FRT his1::FRT/his1::FRT arg4::FRT/arg4::FRT CSE4/CSE4::CSE4-TAP (CaHIS1) sch9::FRT/sch9::FRT Chr5 monosomy Transformant 3 |
| CtKG104 | ura3::FRT/ura3::FRT his1::FRT/his1::FRT arg4::FRT/arg4::FRT CSE4/CSE4::CSE4-TAP (CaHIS1) sch9::FRT/sch9::FRT Chr5 monosomy Transformant 4 |
| CtKG105 | ura3::FRT/ura3::FRT his1::FRT/his1::FRT arg4::FRT/arg4::FRT CSE4/CSE4::CSE4-TAP (CaHIS1) sch9::FRT/sch9::FRT Chr5 monosomy Transformant 5 |
| NCYC-2606 | Environmental isolate of C. sojae (wild-type) |
