# Supplementary material for: Spatial inter-centromeric interactions facilitated the emergence of evolutionary new centromeres
Source: eLife. 2020 May 29;9:e58556. doi: 10.7554/eLife.58556 (PMC7292649; doi:10.7554/eLife.58556)
Supplement: Supplementary file 9. [file elife-58556-supp9.pptx]

## Slide 1
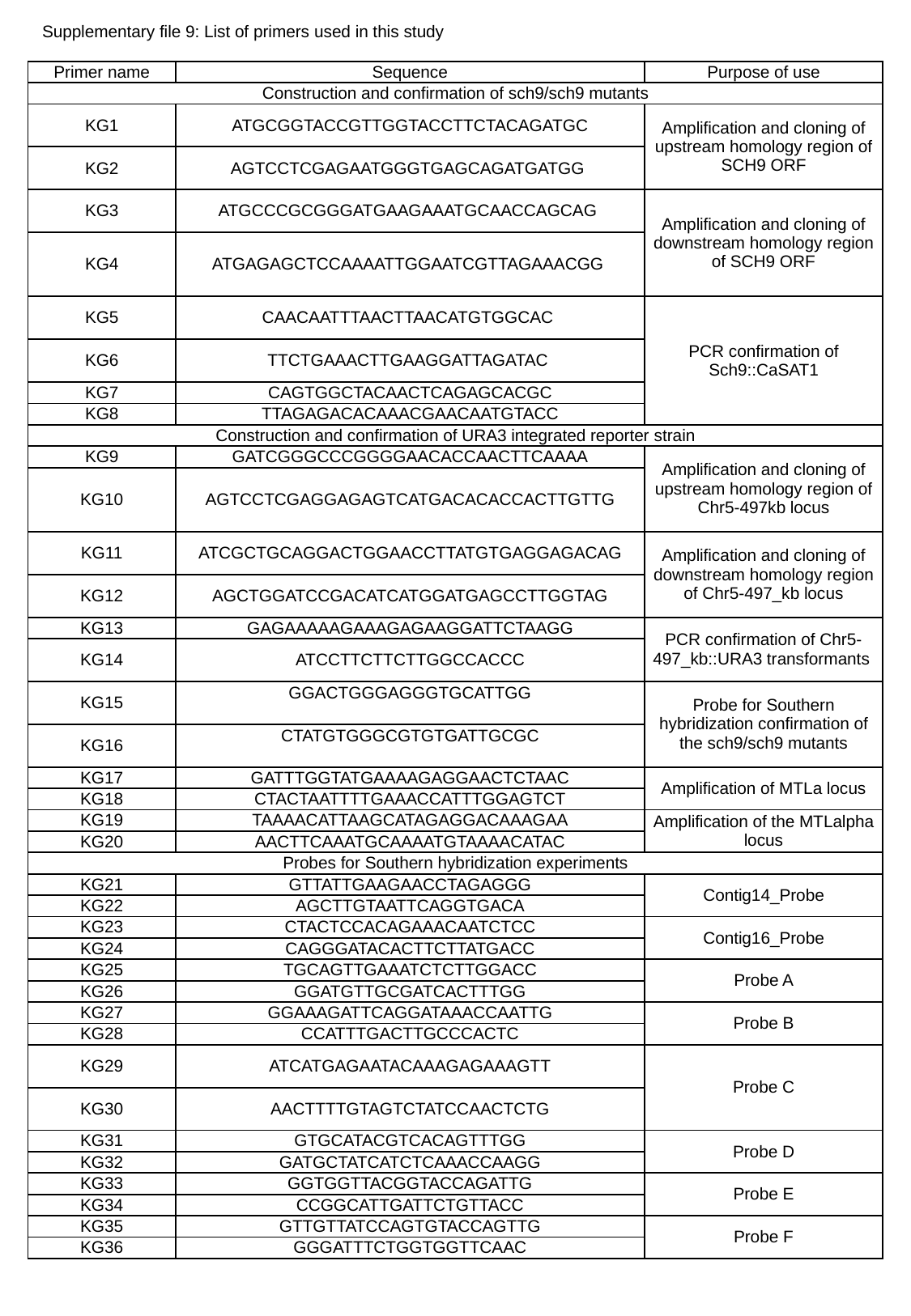

Supplementary file 9: List of primers used in this study
| Primer name | Sequence | Purpose of use |
| --- | --- | --- |
| Construction and confirmation of sch9/sch9 mutants | | |
| KG1 | ATGCGGTACCGTTGGTACCTTCTACAGATGC | Amplification and cloning of upstream homology region of SCH9 ORF |
| KG2 | AGTCCTCGAGAATGGGTGAGCAGATGATGG | |
| KG3 | ATGCCCGCGGGATGAAGAAATGCAACCAGCAG | Amplification and cloning of downstream homology region of SCH9 ORF |
| KG4 | ATGAGAGCTCCAAAATTGGAATCGTTAGAAACGG | |
| KG5 | CAACAATTTAACTTAACATGTGGCAC | PCR confirmation of Sch9::CaSAT1 |
| KG6 | TTCTGAAACTTGAAGGATTAGATAC | |
| KG7 | CAGTGGCTACAACTCAGAGCACGC | |
| KG8 | TTAGAGACACAAACGAACAATGTACC | |
| Construction and confirmation of URA3 integrated reporter strain | | |
| KG9 | GATCGGGCCCGGGGAACACCAACTTCAAAA | Amplification and cloning of upstream homology region of Chr5-497kb locus |
| KG10 | AGTCCTCGAGGAGAGTCATGACACACCACTTGTTG | |
| KG11 | ATCGCTGCAGGACTGGAACCTTATGTGAGGAGACAG | Amplification and cloning of downstream homology region of Chr5-497\_kb locus |
| KG12 | AGCTGGATCCGACATCATGGATGAGCCTTGGTAG | |
| KG13 | GAGAAAAAGAAAGAGAAGGATTCTAAGG | PCR confirmation of Chr5-497\_kb::URA3 transformants |
| KG14 | ATCCTTCTTCTTGGCCACCC | |
| KG15 | GGACTGGGAGGGTGCATTGG | Probe for Southern hybridization confirmation of the sch9/sch9 mutants |
| KG16 | CTATGTGGGCGTGTGATTGCGC | |
| KG17 | GATTTGGTATGAAAAGAGGAACTCTAAC | Amplification of MTLa locus |
| KG18 | CTACTAATTTTGAAACCATTTGGAGTCT | |
| KG19 | TAAAACATTAAGCATAGAGGACAAAGAA | Amplification of the MTLalpha locus |
| KG20 | AACTTCAAATGCAAAATGTAAAACATAC | |
| Probes for Southern hybridization experiments | | |
| KG21 | GTTATTGAAGAACCTAGAGGG | Contig14\_Probe |
| KG22 | AGCTTGTAATTCAGGTGACA | |
| KG23 | CTACTCCACAGAAACAATCTCC | Contig16\_Probe |
| KG24 | CAGGGATACACTTCTTATGACC | |
| KG25 | TGCAGTTGAAATCTCTTGGACC | Probe A |
| KG26 | GGATGTTGCGATCACTTTGG | |
| KG27 | GGAAAGATTCAGGATAAACCAATTG | Probe B |
| KG28 | CCATTTGACTTGCCCACTC | |
| KG29 | ATCATGAGAATACAAAGAGAAAGTT | Probe C |
| KG30 | AACTTTTGTAGTCTATCCAACTCTG | |
| KG31 | GTGCATACGTCACAGTTTGG | Probe D |
| KG32 | GATGCTATCATCTCAAACCAAGG | |
| KG33 | GGTGGTTACGGTACCAGATTG | Probe E |
| KG34 | CCGGCATTGATTCTGTTACC | |
| KG35 | GTTGTTATCCAGTGTACCAGTTG | Probe F |
| KG36 | GGGATTTCTGGTGGTTCAAC | |
