# Supplementary material for: Spatial inter-centromeric interactions facilitated the emergence of evolutionary new centromeres
Source: eLife. 2020 May 29;9:e58556. doi: 10.7554/eLife.58556 (PMC7292649; doi:10.7554/eLife.58556)
Supplement: Supplementary file 10. [file elife-58556-supp10.pptx]

## Slide 1
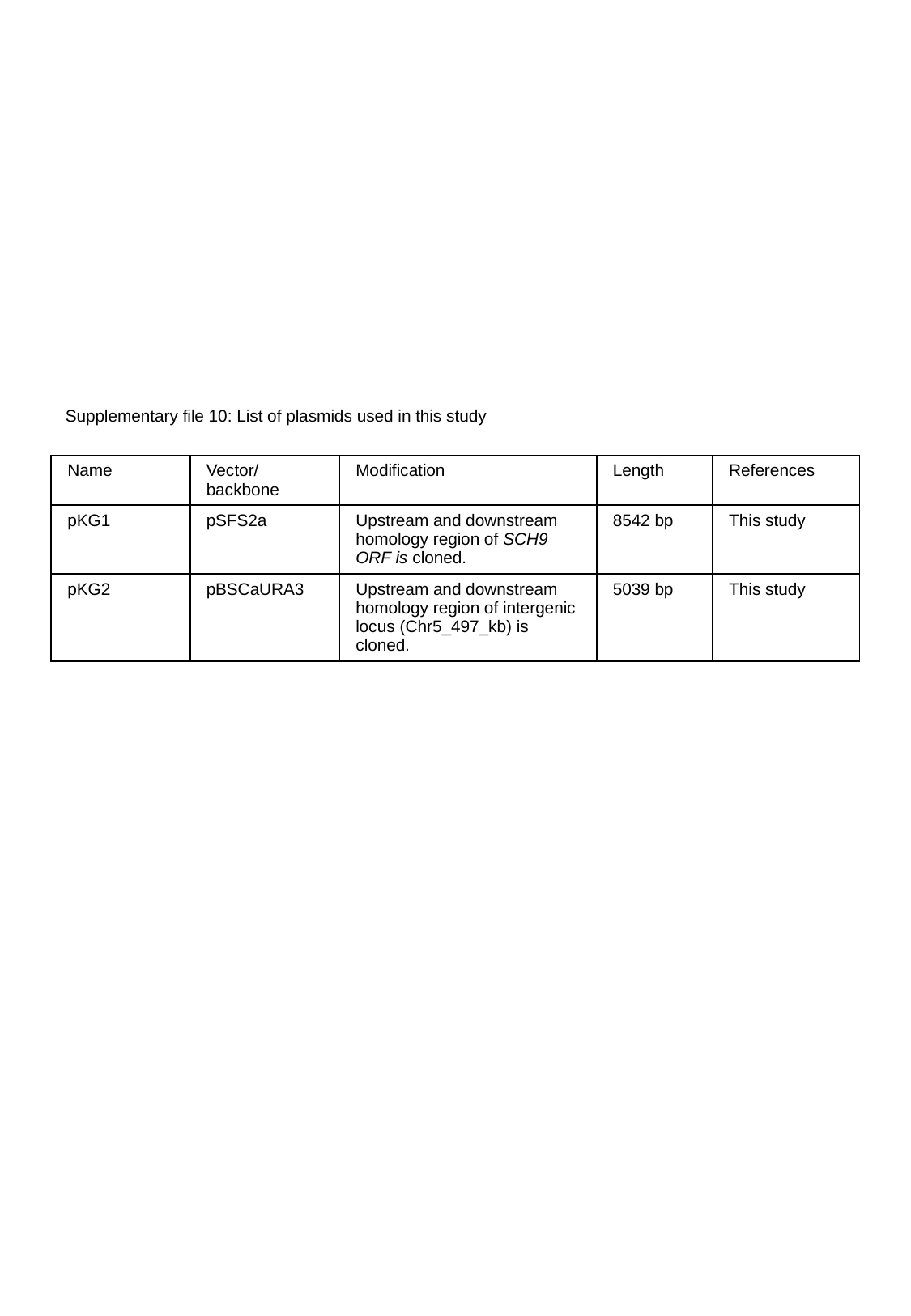

Supplementary file 10: List of plasmids used in this study
| Name | Vector/ backbone | Modification | Length | References |
| --- | --- | --- | --- | --- |
| pKG1 | pSFS2a | Upstream and downstream homology region of SCH9 ORF is cloned. | 8542 bp | This study |
| pKG2 | pBSCaURA3 | Upstream and downstream homology region of intergenic locus (Chr5\_497\_kb) is cloned. | 5039 bp | This study |
